# Triaqua­chlorido(1,10-phenanthroline-κ^2^ *N*,*N*′)cobalt(II) chloride monohydrate

**DOI:** 10.1107/S1600536808042591

**Published:** 2008-12-20

**Authors:** Yaru Liu, Yutian Pan, Junshan Sun, Chuan Zhang

**Affiliations:** a School of Science, North University of China, Taiyuan 030051, People’s Republic of China; b School of Mechatronic Engineering, North University of China, Taiyuan 030051, People’s Republic of China; c Department of Materials Science and Chemical Engineering, Taishan University, 271021 Taian, Shandong, People’s Republic of China

## Abstract

In the title compound, [CoCl(C_12_H_8_N_2_)(H_2_O)_3_]Cl·H_2_O, the Co^II^ ion is coordinated by two N atoms from the 1,10-phenanthroline ligand [Co—N = 2.125 (6) and 2.146 (6) Å], one chloride ligand [Co—Cl = 2.459 (2)Å] and three water mol­ecules [Co—O = 2.070 (5)–2.105 (5)Å] in a distorted octa­hedral geometry. Inter­molecular O—H⋯Cl and O—H⋯O hydrogen bonds form an extensive three-dimensional hydrogen-bonding network, which consolidates the crystal packing.

## Related literature

For related crystal structures, see: Liu *et al.* (2006 ▶); Zhang *et al.* (1999 ▶).
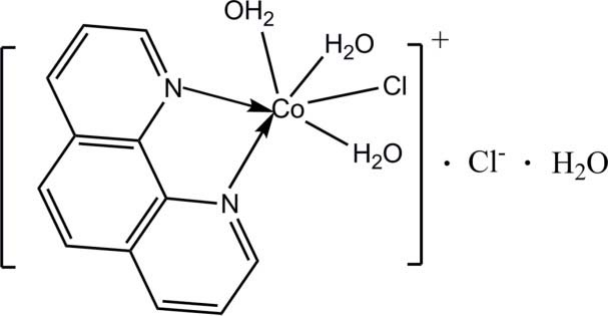


## Experimental

### 

#### Crystal data

[CoCl(C_12_H_8_N_2_)(H_2_O)_3_]Cl·H_2_O
*M* *_r_* = 382.10
Monoclinic, 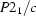

*a* = 7.646 (4) Å
*b* = 12.426 (6) Å
*c* = 16.987 (8) Å
β = 103.54 (2)°
*V* = 1569.1 (13) Å^3^
*Z* = 4
Mo *K*α radiation
μ = 1.45 mm^−1^
*T* = 273 (2) K
0.37 × 0.25 × 0.19 mm

#### Data collection

Bruker SMART CCD area-detector diffractometer
Absorption correction: multi-scan (*SADABS*; Sheldrick, 1996 ▶) *T*_min_ = 0.616, *T*_max_ = 0.770
6533 measured reflections
2733 independent reflections
1564 reflections with *I* > 2σ(*I*)
*R*_int_ = 0.080

#### Refinement

*R*[*F* ^2^ > 2σ(*F* ^2^)] = 0.071
*wR*(*F* ^2^) = 0.177
*S* = 1.04
2733 reflections
198 parameters
13 restraints
H-atom parameters constrained
Δρ_max_ = 0.67 e Å^−3^
Δρ_min_ = −0.56 e Å^−3^

### 

Data collection: *SMART* (Siemens, 1996 ▶); cell refinement: *SAINT* (Siemens, 1996 ▶); data reduction: *SAINT*; program(s) used to solve structure: *SHELXS97* (Sheldrick, 2008 ▶); program(s) used to refine structure: *SHELXL97* (Sheldrick, 2008 ▶); molecular graphics: *SHELXTL* (Sheldrick, 2008 ▶); software used to prepare material for publication: *SHELXTL*.

## Supplementary Material

Crystal structure: contains datablocks I, global. DOI: 10.1107/S1600536808042591/cv2481sup1.cif

Structure factors: contains datablocks I. DOI: 10.1107/S1600536808042591/cv2481Isup2.hkl

Additional supplementary materials:  crystallographic information; 3D view; checkCIF report

## Figures and Tables

**Table 1:** Hydrogen-bond geometry (Å, °)

*D*—H⋯*A*	*D*—H	H⋯*A*	*D*⋯*A*	*D*—H⋯*A*
O1—H1*A*⋯O4	0.85	1.93	2.693 (9)	149
O1—H1*B*⋯Cl2	0.85	2.55	3.258 (6)	141
O2—H2*A*⋯Cl1^i^	0.85	2.31	3.139 (6)	166
O2—H2*B*⋯Cl2^i^	0.85	2.29	3.119 (6)	164
O3—H3*A*⋯Cl2^ii^	0.85	2.33	3.114 (6)	153
O3—H3*B*⋯Cl1^ii^	0.85	2.30	3.128 (6)	166
O4—H4*A*⋯Cl2^iii^	0.85	2.34	3.185 (8)	170
O4—H4*B*⋯Cl1^ii^	0.85	2.58	3.278 (8)	141

Symmetry codes: (i) 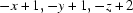; (ii) 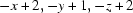; (iii) 
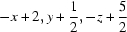
.

**Fig. 1.**
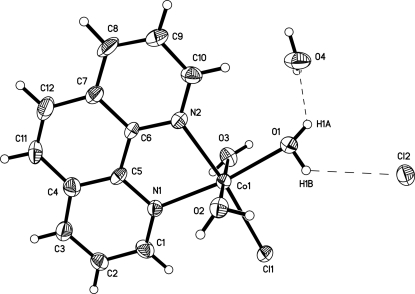
The structure of the title complex, showing 30% probability displacement ellipsoids and the atom-numbering scheme. Dashed lines denote hydrogen bonds.

## supplementary crystallographic information

### Comment

The structures of some tetraaqua(1,10-phenanthroline) metal ionic complexes with different anions have been reported (Liu *et al.*, 2006; Zhang *et al.*, 1999). In our study in this field, we select 1,10-phenanthroline as the co-ligand to continue our exploration of the Co complexes. Herein we report the structure of the title compound (I), which was characterized by elemental analyses and X-ray crystallography diffraction.

In (I) (Fig. 1), each Co^II^ ion is coordinated by two N atoms from 1,10-phenanthroline ligand [Co—N 2.125 (6), 2.146 (6) Å], one chlorine anion [Co—Cl 2.459 (2) Å] and three water molecules [Co—O 2.070 (5)–2.105 (5) Å] in a distorted octahedral geometry. Two aqua O atoms and two N atoms from 1,10-phenanthroline ligand define the special position on a mirror plane. One aqua group and chlorine anion  fill  the axial apical positions. The intermolecular O—H···Cl and O—H···O hydrogen bonds (Table 1) form an extensive three-dimensional hydrogen-bonding network, which consolidate the crystal packing.

### Experimental

A mixture of Cu(Cl)2*3H2O(168 mg, 1 mmol) and 1,10-phenanthroline(185 mg, 1 mmol) in methanol(30 ml) was placed in a Teflon-lined stainless steel Parr bomb that was heated at 423 K for 72 h. The bomb was then cooled down to the room temperature, the solution was filtered. The solvent was removed from the filtrate under vacuum, and the solid residue was recrystallized from diethyl ether; blue crystals suitable for X-Ray diffraction study were obtained. Yield, 0.724 g, 81%. m.p. 566 K. Analysis, calculated for C_12_H_16_Cl_2_CoN_2_O_4_: C 37.72, H 4.22, N 7.33; found: C 37.53, H 4.68, N 7.17%. The elemental analyses were performed with a Perkine Elemer PE2400II instrument.

### Refinement

Water H atoms were located in a differece map and were isotropically refined with an O—H strict distance restraint of 0.85/%A. H atoms bound to C atoms were placed in calculated positions(C—H = 0.93/%A) and refined in the riding-model approximation, *U*_iso_(H)=1.2*U*_eq_(C).

### Crystal data

**Table:**

[CoCl(C_12_H_8_N_2_)(H_2_O)_3_]Cl·H_2_O	*F*(000) = 780
*M**_r_* = 382.10	*D*_x_ = 1.617 Mg m^−^^3^
Monoclinic, *P*2_1_/*c*	Mo *K*α radiation, λ = 0.71073 Å
*a* = 7.646 (4) Å	Cell parameters from 580 reflections
*b* = 12.426 (6) Å	θ = 3.0–20.1°
*c* = 16.987 (8) Å	µ = 1.45 mm^−^^1^
β = 103.54 (2)°	*T* = 273 K
*V* = 1569.1 (13) Å^3^	Block, blue
*Z* = 4	0.37 × 0.25 × 0.19 mm

### Data collection

**Table:**

Bruker SMART CCD area-detector diffractometer	2733 independent reflections
Radiation source: fine-focus sealed tube	1564 reflections with *I* > 2σ(*I*)
graphite	*R*_int_ = 0.080
φ and ω scans	θ_max_ = 25.0°, θ_min_ = 2.1°
Absorption correction: multi-scan (*SADABS*; Sheldrick, 1996)	*h* = −9→5
*T*_min_ = 0.616, *T*_max_ = 0.770	*k* = −10→14
6533 measured reflections	*l* = −17→20

### Refinement

**Table:**

Refinement on *F*^2^	Primary atom site location: structure-invariant direct methods
Least-squares matrix: full	Secondary atom site location: difference Fourier map
*R*[*F*^2^ > 2σ(*F*^2^)] = 0.071	Hydrogen site location: inferred from neighbouring sites
*wR*(*F*^2^) = 0.177	H-atom parameters constrained
*S* = 1.04	*w* = 1/[σ^2^(*F*_o_^2^) + (0.0517*P*)^2^ + 4.0479*P*] where *P* = (*F*_o_^2^ + 2*F*_c_^2^)/3
2733 reflections	(Δ/σ)_max_ < 0.001
198 parameters	Δρ_max_ = 0.67 e Å^−^^3^
13 restraints	Δρ_min_ = −0.56 e Å^−^^3^

### Special details

**Table:**

Geometry. All e.s.d.'s (except the e.s.d. in the dihedral angle between two l.s. planes) are estimated using the full covariance matrix. The cell e.s.d.'s are taken into account individually in the estimation of e.s.d.'s in distances, angles and torsion angles; correlations between e.s.d.'s in cell parameters are only used when they are defined by crystal symmetry. An approximate (isotropic) treatment of cell e.s.d.'s is used for estimating e.s.d.'s involving l.s. planes.
Refinement. Refinement of *F*^2^ against ALL reflections. The weighted *R*-factor *wR* and goodness of fit *S* are based on *F*^2^, conventional *R*-factors *R* are based on *F*, with *F* set to zero for negative *F*^2^. The threshold expression of *F*^2^ > σ(*F*^2^) is used only for calculating *R*-factors(gt) *etc*. and is not relevant to the choice of reflections for refinement. *R*-factors based on *F*^2^ are statistically about twice as large as those based on *F*, and *R*- factors based on ALL data will be even larger.

### Fractional atomic coordinates and isotropic or equivalent isotropic displacement parameters (Å^2^)

**Table:**

	*x*	*y*	*z*	*U*_iso_*/*U*_eq_	
Co1	0.75359 (13)	0.66886 (9)	0.99070 (6)	0.0342 (3)	
Cl1	0.6997 (3)	0.49910 (16)	0.91423 (11)	0.0431 (5)	
Cl2	0.8145 (3)	0.34056 (18)	1.16347 (12)	0.0536 (6)	
N1	0.6918 (8)	0.7697 (5)	0.8867 (4)	0.0360 (15)	
N2	0.7887 (8)	0.8255 (5)	1.0452 (4)	0.0405 (16)	
O1	0.8190 (8)	0.5892 (4)	1.1032 (3)	0.0478 (15)	
H1A	0.9003	0.5920	1.1469	0.15 (6)*	
H1B	0.7797	0.5251	1.0958	0.10 (4)*	
O2	0.4845 (7)	0.6729 (4)	0.9965 (3)	0.0459 (13)	
H2A	0.4527	0.6214	1.0231	0.06 (3)*	
H2B	0.4185	0.6755	0.9488	0.05 (3)*	
O3	1.0230 (7)	0.6657 (4)	0.9883 (3)	0.0456 (14)	
H3A	1.0372	0.6788	0.9411	0.05 (3)*	
H3B	1.1121	0.6289	1.0137	0.09 (4)*	
O4	1.1261 (10)	0.6517 (6)	1.2062 (4)	0.075 (2)	
H4A	1.1566	0.7013	1.2412	0.06 (3)*	
H4B	1.2171	0.6296	1.1901	0.16 (7)*	
C1	0.6476 (11)	0.7393 (8)	0.8103 (5)	0.049 (2)	
H1	0.6394	0.6664	0.7979	0.059*	
C2	0.6123 (11)	0.8160 (7)	0.7468 (5)	0.050 (2)	
H2	0.5810	0.7933	0.6931	0.060*	
C3	0.6243 (11)	0.9222 (7)	0.7645 (5)	0.052 (2)	
H3	0.6017	0.9726	0.7228	0.062*	
C4	0.6706 (11)	0.9573 (7)	0.8456 (5)	0.046 (2)	
C5	0.7032 (10)	0.8787 (6)	0.9056 (5)	0.037 (2)	
C6	0.7525 (9)	0.9047 (6)	0.9885 (5)	0.0338 (17)	
C7	0.7636 (10)	1.0150 (7)	1.0107 (5)	0.044 (2)	
C8	0.8142 (12)	1.0398 (8)	1.0931 (6)	0.062 (3)	
H8	0.8232	1.1111	1.1102	0.075*	
C9	0.8501 (13)	0.9583 (8)	1.1481 (6)	0.062 (3)	
H9	0.8841	0.9740	1.2031	0.074*	
C10	0.8363 (10)	0.8527 (7)	1.1225 (5)	0.049 (2)	
H10	0.8616	0.7985	1.1612	0.058*	
C11	0.6849 (12)	1.0667 (7)	0.8682 (6)	0.058 (3)	
H11	0.6645	1.1199	0.8285	0.070*	
C12	0.7284 (13)	1.0944 (7)	0.9475 (6)	0.063 (3)	
H12	0.7357	1.1669	0.9614	0.075*	

### Atomic displacement parameters (Å^2^)

**Table:**

	*U*^11^	*U*^22^	*U*^33^	*U*^12^	*U*^13^	*U*^23^
Co1	0.0399 (6)	0.0288 (6)	0.0335 (6)	−0.0003 (5)	0.0080 (4)	0.0012 (5)
Cl1	0.0576 (13)	0.0299 (11)	0.0394 (12)	−0.0009 (9)	0.0068 (9)	−0.0034 (10)
Cl2	0.0673 (14)	0.0495 (14)	0.0443 (12)	0.0020 (11)	0.0133 (10)	0.0060 (12)
N1	0.040 (4)	0.033 (4)	0.036 (4)	0.000 (3)	0.011 (3)	0.002 (3)
N2	0.043 (4)	0.034 (4)	0.045 (4)	−0.001 (3)	0.009 (3)	−0.003 (4)
O1	0.068 (4)	0.040 (4)	0.034 (3)	−0.006 (3)	0.009 (3)	0.002 (3)
O2	0.050 (3)	0.042 (4)	0.048 (3)	0.000 (3)	0.016 (3)	0.004 (3)
O3	0.043 (3)	0.055 (4)	0.042 (3)	0.006 (3)	0.015 (2)	0.007 (3)
O4	0.080 (5)	0.080 (5)	0.058 (4)	0.008 (4)	0.001 (4)	−0.028 (4)
C1	0.058 (6)	0.059 (6)	0.030 (5)	0.005 (4)	0.010 (4)	0.004 (4)
C2	0.061 (6)	0.055 (6)	0.032 (4)	0.003 (4)	0.008 (4)	0.004 (4)
C3	0.062 (6)	0.040 (6)	0.052 (6)	0.003 (4)	0.012 (4)	0.005 (5)
C4	0.046 (5)	0.044 (6)	0.047 (5)	0.005 (4)	0.009 (4)	0.006 (4)
C5	0.036 (4)	0.025 (4)	0.055 (6)	0.000 (3)	0.018 (4)	−0.009 (4)
C6	0.038 (4)	0.022 (4)	0.042 (5)	−0.001 (3)	0.010 (3)	−0.001 (4)
C7	0.043 (5)	0.032 (5)	0.057 (6)	0.001 (4)	0.013 (4)	−0.005 (4)
C8	0.078 (7)	0.034 (6)	0.074 (7)	−0.002 (5)	0.014 (5)	−0.023 (5)
C9	0.080 (7)	0.045 (6)	0.059 (6)	−0.001 (5)	0.016 (5)	−0.021 (5)
C10	0.053 (5)	0.054 (6)	0.038 (5)	0.004 (4)	0.009 (4)	−0.004 (5)
C11	0.072 (7)	0.038 (6)	0.062 (7)	0.009 (5)	0.012 (5)	0.020 (5)
C12	0.075 (7)	0.030 (5)	0.082 (7)	0.002 (5)	0.013 (5)	0.004 (6)

### Geometric parameters (Å, °)

**Table:**

Co1—O3	2.071 (5)	C1—H1	0.9300
Co1—O2	2.084 (5)	C2—C3	1.35 (1)
Co1—O1	2.106 (5)	C2—H2	0.9301
Co1—N1	2.127 (6)	C3—C4	1.41 (1)
Co1—N2	2.145 (7)	C3—H3	0.9300
Co1—Cl1	2.461 (2)	C4—C5	1.39 (1)
N1—C1	1.317 (9)	C4—C11	1.41 (1)
N1—C5	1.390 (9)	C5—C6	1.41 (1)
N2—C10	1.321 (9)	C6—C7	1.42 (1)
N2—C6	1.361 (9)	C7—C8	1.40 (1)
O1—H1A	0.8500	C7—C12	1.44 (1)
O1—H1B	0.8501	C8—C9	1.36 (1)
O2—H2A	0.8500	C8—H8	0.9301
O2—H2B	0.8498	C9—C10	1.38 (1)
O3—H3A	0.8500	C9—H9	0.9301
O3—H3B	0.8500	C10—H10	0.9300
O4—H4A	0.8500	C11—C12	1.36 (1)
O4—H4B	0.8500	C11—H11	0.9300
C1—C2	1.42 (1)	C12—H12	0.9300
			
O3—Co1—O2	178.4 (2)	C1—C2—H2	120.2
O3—Co1—O1	89.0 (2)	C2—C3—C4	120.5 (8)
O2—Co1—O1	89.7 (2)	C2—C3—H3	119.7
O3—Co1—N1	91.3 (2)	C4—C3—H3	119.7
O2—Co1—N1	89.8 (2)	C5—C4—C3	117.4 (8)
O1—Co1—N1	171.9 (2)	C5—C4—C11	119.3 (8)
O3—Co1—N2	90.1 (2)	C3—C4—C11	123.3 (8)
O2—Co1—N2	89.0 (2)	N1—C5—C4	121.6 (8)
O1—Co1—N2	93.2 (2)	N1—C5—C6	116.3 (7)
N1—Co1—N2	78.8 (2)	C4—C5—C6	122.0 (7)
C1—N1—C5	119.6 (7)	N2—C6—C5	120.3 (7)
C1—N1—Co1	127.3 (6)	N2—C6—C7	121.4 (7)
C5—N1—Co1	113.1 (5)	C5—C6—C7	118.3 (7)
C10—N2—C6	118.8 (7)	C8—C7—C6	117.7 (8)
C10—N2—Co1	129.7 (6)	C8—C7—C12	123.8 (8)
C6—N2—Co1	111.5 (5)	C6—C7—C12	118.4 (8)
Co1—O1—H1A	138.0	C9—C8—C7	119.2 (8)
Co1—O1—H1B	107.8	C9—C8—H8	120.4
H1A—O1—H1B	109.2	C7—C8—H8	120.4
Co1—O2—H2A	114.5	C8—C9—C10	120.2 (9)
Co1—O2—H2B	109.1	C8—C9—H9	119.9
H2A—O2—H2B	110.8	C10—C9—H9	119.9
Co1—O3—H3A	111.3	N2—C10—C9	122.7 (9)
Co1—O3—H3B	133.1	N2—C10—H10	118.7
H3A—O3—H3B	108.6	C9—C10—H10	118.7
H4A—O4—H4B	110.4	C12—C11—C4	120.0 (8)
N1—C1—C2	121.2 (8)	C12—C11—H11	120.0
N1—C1—H1	119.4	C4—C11—H11	120.0
C2—C1—H1	119.4	C11—C12—C7	121.9 (9)
C3—C2—C1	119.7 (8)	C11—C12—H12	119.1
C3—C2—H2	120.2	C7—C12—H12	119.1

### Hydrogen-bond geometry (Å, °)

**Table:**

*D*—H···*A*	*D*—H	H···*A*	*D*···*A*	*D*—H···*A*
O1—H1A···O4	0.85	1.93	2.693 (9)	149
O1—H1B···Cl2	0.85	2.55	3.258 (6)	141
O2—H2A···Cl1^i^	0.85	2.31	3.139 (6)	166
O2—H2B···Cl2^i^	0.85	2.29	3.119 (6)	164
O3—H3A···Cl2^ii^	0.85	2.33	3.114 (6)	153
O3—H3B···Cl1^ii^	0.85	2.30	3.128 (6)	166
O4—H4A···Cl2^iii^	0.85	2.34	3.185 (8)	170
O4—H4B···Cl1^ii^	0.85	2.58	3.278 (8)	141

Symmetry codes: (i) −*x*+1, −*y*+1, −*z*+2; (ii) −*x*+2, −*y*+1, −*z*+2; (iii) −*x*+2, *y*+1/2, −*z*+5/2.
